# Illuminating the Shadows of Neuroscience: How Curiosity and Courage Redefine Research Models and Mentorship

**DOI:** 10.1523/ENEURO.0056-25.2025

**Published:** 2025-04-25

**Authors:** Kelly G. Lambert

**Affiliations:** Department of Psychology, University of Richmond, Richmond, Virginia 23173

**Keywords:** experience-based neuroplasticity, mentoring, neuroethology, neuroscience research models, translational research

## Abstract

Scientific progress often hinges on the courage to question conventional models and embrace exploratory research. This commentary examines the importance of curiosity-driven science by drawing on historical and contemporary examples, from Darwin's unconventional research methodologies to modern neuroscience investigations that challenge the constraints of traditional laboratory environments. An over-reliance on predictable, controlled conditions—particularly in select rodent models—has potentially limited the translational impact of neuroscience. By exploring novel research paradigms, including raccoon neural investigations and rodent-driven vehicles (ROVs), the value of expanding research models to incorporate diverse species and environments is examined. As neuroscience advances, the field must move beyond the metaphorical lamp post, venturing into uncharted territory to fully capture the complexity of neurobiological variables. By fostering a culture of discovery over predictability—supported by innovative and exploratory mentoring approaches—we can illuminate new frontiers in neuroscience and develop transformative insights for mental health and beyond.

## Significance Statement

Despite impressive advances in neuroscience, translational efficacy for psychiatric treatments remains low. An overreliance on selectively bred rodents housed in featureless environments may limit generalizability to real-world mental health contexts. Historically, unconventional research approaches have yielded transformative discoveries. Research in our laboratory reinforces the importance of investigating diverse animal models in varying forms of enriched environments to capture a fuller spectrum of brain plasticity and behavior. Expanding neuroscience training programs to incorporate naturalistic models and ecologically relevant settings has the potential to improve translational success, offering novel insights into cognitive flexibility and emotional regulation. To advance neuroscience's impact on mental health, students and trainees should engage with diverse research models, environments, and methodologies—fostering a comprehensive understanding of brain function.

## Lessons from Darwin's Research Approach

Darwin's voyage aboard the HMS Beagle is a curiosity-driven odyssey that often feels more like fiction than the foundation of modern biological science. Lacking an advanced degree or a formal faculty position, he embarked on a 5-year journey, meticulously collecting observations and specimens that would ultimately upend the dominant religious narratives of creation. Yet, despite the significance of his findings, Darwin delayed publishing, fearing professional backlash and personal conflict—particularly with his devout wife, Emma. It was only the looming prospect of being preempted by Alfred Russel Wallace that finally spurred him to release *On the Origin of Species* in 1859 (Darwin, 1964; Boulter, 2009).

In a poignant letter to his friend Joseph Hooker, Darwin described the unveiling of his theory of natural selection as akin to “confessing a murder” (Barlow, 1958). This striking metaphor reflects the emotional weight of challenging entrenched beliefs—scientific, societal, religious, and personal. To clear his thoughts and manage his anxiety, Darwin developed a routine of walking laps around his “thinking path” at Down House, a practice that blended nature, exercise, and quiet reflection—an early illustration of what we might now call behavior-based therapeutics (Boulter, 2009; Merzenich et al., 2014). In my lab, we refer to these behavioral interventions as *behaviorceuticals*, and Darwin's approach serves as a fascinating case study (Lambert, 2018). Recognizing his own need for “higher doses” of this walking therapy, he devised a simple yet brilliant system that involved placing a specific number of flint stones at the gate and removing one with each completed lap, freeing his mind from the distraction of counting his self-prescribed laps for the day. Darwin's contributions, however, extended far beyond personal reflections. More than a century later, evolutionary biologist Theodosius Dobzhansky famously asserted, “Nothing in biology makes sense except in the light of evolution” (Dobzhansky, 1973).

Despite our deep appreciation for curiosity-driven pioneers such as Darwin, modern academic structures often suppress the very mindset that made his discoveries possible. Today, funding priorities, tenure expectations, and institutional pressures tend to reward safe, confirmatory research rather than bold, exploratory science. Accordingly, I often wonder if Darwin's curiosity would have survived in the traditional laboratory environments that characterize contemporary research spaces. Or, would the rigid structures of contemporary academia have stifled the very instincts that led him to revolutionize biological science? If we are to inspire the next generation of impactful neuroscientists, we must actively nurture a culture that prioritizes discovery over predictability. Darwin's ability to embrace uncertainty—not just scientific but deeply personal uncertainty—offers a timeless model for advancing knowledge in neuroscience, a field that remains riddled with unanswered questions about the complexity of the brain and mental health.

## The Lamp Post Analogy: Looking Beyond the Familiar

In the well-known lamp post parable, a police officer sees an inebriated man searching under a lamp post and asks what he is looking for. The man replies, “My keys.” When the officer inquires whether he lost them there, the man gestures toward a darker area across the parking lot and admits, “No, but this is where the light is.”

**Figure 1.**
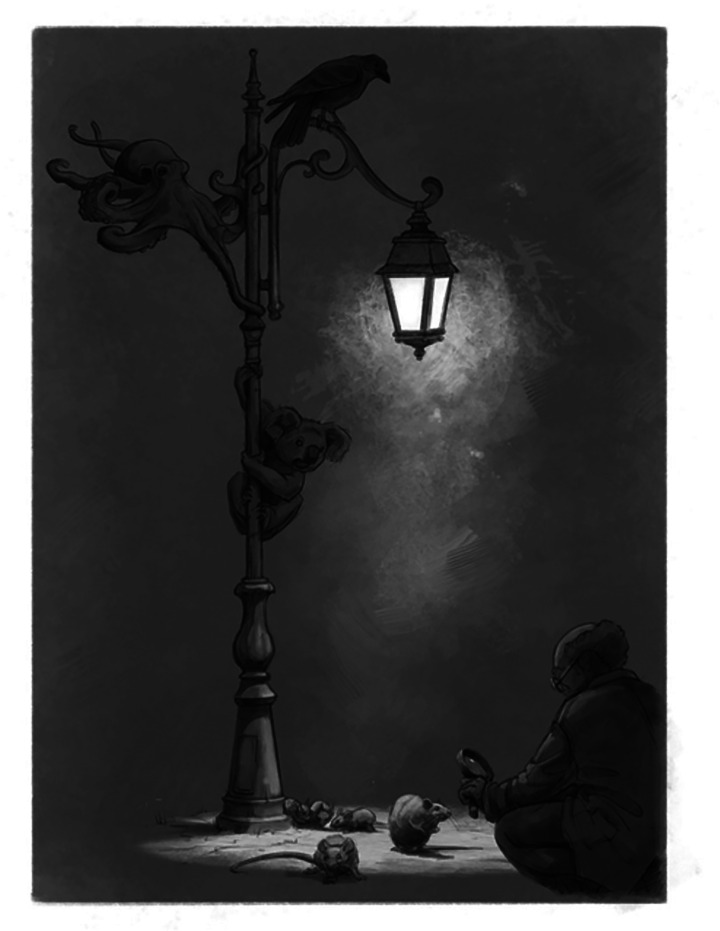
The neuroscience lamp post. Despite the vast diversity of nervous system models in nature, neuroscience research has largely focused on a few rodent models. If neuroscientists look beyond the limited light provided by the laboratory lamp post, diverse species can be observed and investigated. While selectively bred animals in controlled environments offer practical advantages, expanding research to diverse animal models can provide richer insights into brain function. (Illustration by Javier Lazaro, published with the artist's permission.)

Similarly, neuroscientists have long confined their inquiries to the “light” of familiar, controlled conditions. By focusing predominantly on selectively bred rodents housed in artificial environments, researchers risk missing the broader insights offered by the diverse nervous systems that exist in nature. While laboratory research has yielded critical discoveries and impressive Nobel Prizes, it's time to also step beyond the lamp post and embrace the complexity and diversity of diverse nervous systems in natural environments (Fig. 1).

For much of my career, I, too, have conducted research under the metaphorical lamp post—working primarily with selectively bred rodents in standard laboratory environments. These studies remain valuable, but our lab is currently expanding its targeted research models to include captive and wild raccoons, semi-free-ranging macaques, and preliminary investigations of mouse lemurs living in their natural habitat. In our rodent studies, we are now comparing wild-trapped and laboratory-bred *Rattus norvegicus*. Despite living just miles apart, wild and lab rats have dramatically different adaptations—most notably related to their stress response. We have reported urban wild rats, for example, to have fecal corticosterone metabolite levels approximately six times higher than their lab counterparts (Jacob et al., 2022).

If laboratory rats fail to reliably generalize to wild rats of the same species, how can we expect laboratory rodent findings to translate to humans? Behavioral scientists have acknowledged the limitations of investigating predominantly western, educated, industrialized, rich, and democratic (WEIRD) human populations and have taken steps to address this bias (Henrich et al., 2010). Neuroscience research is due for a similar reckoning. Overreliance on a handful of model species limits the translational value of our research. Further, the limitation of restricted laboratory habitats is underscored by recent findings suggesting that laboratory rats in traditional isolated housing experience accelerated morbidity, with increased risks of cardiovascular disease, cancer, stroke, and heightened anxiety (Cait et al., 2022). These conditions likely distort baseline health and behavioral findings in ways we have yet to fully appreciate.

Reflecting on my personal research trajectory, I often describe myself as a recovering Laboratory Animal Model Exclusive (LAME) researcher. While traditional rodent models have provided invaluable insights, neuroscience needs to expand its animal model repertoire. Stepping beyond the lamp post will offer fresh perspectives on nervous system functions, advancing our understanding of the brain, as well as our ability to translate findings to human health.

## Embracing Uncertainty and Doubt

In his essay entitled *The Value of Science*, Nobel laureate and theoretical physicist Richard Feynman championed doubt and uncertainty as essential forces in scientific inquiry. He argued that it is better to confront unanswered questions than to cling to false or unchallengeable answers (Feynman, 1955). His insights proved prescient during the 1986 Challenger disaster investigation, where he famously demonstrated the failure of the rubber O-rings under varying temperatures—exposing a critical flaw that could have prevented the tragedy if the right questions had been asked (Feynman, 1988). Just as engineering failures have highlighted the dangers of disregarding uncertainty, neuroscience progress risks stagnation when adhering too rigidly to established models overlooks alternative approaches. Uncertainty, once a cornerstone of scientific progress, is increasingly viewed as an obstacle rather than a guiding principle in research.

Expanding neuroscience research beyond conventional models seems like a logical step, but it introduces methodological and logistical challenges. Take the raccoon, for example. If scaled to human size, the raccoon's brain exhibits a neuronal density comparable with humans (∼86 billion neurons; Jardim-Messeder et al., 2017). In contrast, a scaled-up rat brain would contain only ∼12 billion neurons—far fewer than observed in humans (Herculano-Houzel, 2009). Further, fast-producing von Economo neurons that haven’t been reliably identified in rodent brains (Raghanti et al., 2015) have been documented in the raccoon brain (Jacob et al., 2021). These findings suggest that raccoons could offer valuable insights into human cognition. Yet, the absence of even a basic raccoon brain atlas—essential for fundamental neuroanatomy—illustrates the systemic barriers to pursuing nontraditional models.

For early-career scientists navigating competitive tenure and promotion systems, the challenges associated with novel research models can be particularly daunting. Institutional and funding barriers often hinder the adoption of novel research models, potentially delaying transformative discoveries. Feynman's curiosity-driven philosophy is especially relevant in today's neuroscience research climate, where the industrial complex of rodent research persists despite the limitations of these models.

While pressure to adhere to traditional research models remains strong, scientists have successfully explored less commonly studied species. Consequently, these alternative models are transforming our understanding of neurobiological functions and mechanisms, challenging traditional laboratory approaches. Naked mole rats, for example, thrive for over three decades and exhibit remarkable resistance to aging and neurodegeneration, offering an alternative model to the short-lived laboratory mice that remain the dominant model for developmental and aging research (Buffenstein et al., 2022). Common shrews offer an equally fascinating perspective—these small mammals digest ∼20% of their brain tissue during harsh winters and regrow it when food is abundant, providing a unique model for neural repair and plasticity (Lázaro et al., 2018). Octopuses, with their decentralized nervous system extending into their arms, challenge mammalian-centric views of cognition and problem-solving (Carls-Diamante, 2022). Similarly, corvids (crows, ravens, and jays) demonstrate exceptional cognitive flexibility, rivaling primates in problem-solving and tool use (Seed et al., 2009). At the other end of the neural adaptation spectrum, koalas exhibit extreme cognitive rigidity, likely linked to their relatively small brain size. Koalas illustrate the evolutionary trade-offs of specialization as they have developed a highly specialized lifestyle focused on the consumption of poisonous eucalyptus leaves (Roger and Handasyde, 1999).

The prairie vole exemplifies how studying diverse species in their natural habitats can transform neurobiological research strategies. Wild prairie voles were initially trapped in monogamous pairs, offering an early clue to their distinctive social structure. Since those serendipitous observations, prairie voles have revolutionized research on social bonding and oxytocin's role in neurobiology, providing critical insights into the mechanisms underlying attachment and affiliative behaviors (Getz et al., 1981; Carter et al., 1995). Observing these voles in their natural habitat revealed a rich model for monogamous and biparental behaviors—patterns absent in standard laboratory mice and rats (Lambert and Byrnes, 2019). Prairie voles serve as a reminder that, while nervous systems share conserved characteristics, selection pressures sculpt adaptations that uniquely equip each species to thrive in its ecological niche. Collectively, these diverse nervous system models underscore the need to recognize the complexity of nervous systems and challenge assumptions associated with the use of standardized research models.

## Curiosity-Driven Research in Action: Rat Driving and Positive Emotions

“What are you scared of, Kelly?” This simple question, posed by the late Jaak Panksepp during a visit to my lab several years ago, has lingered in my mind ever since. Sensing my hesitation to move beyond traditional behavioral assessments, he encouraged me to embrace nontraditional, yet relevant, research approaches. Panksepp, renowned for his pioneering research on “laughing rats,” exemplified the courage required to deviate from conventional paths in rodent emotion research (Panksepp and Burgdorf, 2003). His landmark book, *Affective Neuroscience*, helped illuminate the shadows of neuroscience by exploring the emotional lives of nonhuman animals—an area that had long been overlooked (Panksepp, 2004).

Darwin also recognized the profound role of emotions in animals, as documented in *The Expression of Emotions in Man and Animals* (Darwin, 1872). However, following this landmark publication, scientific interest in emotional influences on neurobiological circuits faded. Although modern neuroscience often emphasizes fear, stress, and aggression, the broader spectrum of emotions—including positive emotions such as seeking and joy, as described by Panksepp, has been largely ignored.

I recalled Panksepp's words when a colleague, cognitive scientist Beth Crawford, asked if I could teach a rat to drive a car. My traditional training urged caution, warning me against pursuing a project that might be dismissed as whimsical. Yet, Panksepp's lingering voice nudged me toward curiosity, challenging me to push past conventional wisdom. As a mentor of undergraduate neuroscience students, I recognized that this unconventional research project could captivate their interest and serve as a gateway to deeper engagement with behavioral neuroscience. Although my lab typically prioritizes ecologically relevant investigations, the rat-driving project presented a unique opportunity to explore skill acquisition and cognitive adaptability in a widely studied mammalian model. What began as a seemingly lighthearted question quickly evolved into a serious investigation of learning, cognitive flexibility, and embodied cognition. After all, mobility—whether in humans or other animals—is shaped by adaptability and exploration. Suddenly, the investigation of how a nonhuman species engages with a novel mode of movement and transportation strategies seemed like a valuable research question.

Nearly a decade later, the driving project has far exceeded my expectations. Using fundamental operant conditioning techniques, students trained rats to approach cars, activate steering mechanisms, and operate rodent-driven vehicles (ROVs) to navigate toward a goal and earn coveted Froot Loop rewards. Our initial findings revealed that rats raised in enriched environments mastered driving skills faster than those from impoverished conditions (Crawford et al., 2020). Despite my initial anxiety about how this research would be received, it has since become a cornerstone of both research and science outreach in my laboratory.

The driving rats have also captured public interest, providing opportunities for public engagement with neuroscience content. Within a month of publication in 2020 (Crawford et al., 2020), over 1,500 media stories covered the study, and this momentum continued in 2024 following an essay in *The Conversation* (Lambert, 2024). The driving rat research has since been featured in the Netflix documentary *The Hidden Lives of Pets*, the Canadian Broadcasting Corporation documentary *Rat City*, and even a National Public Radio-sponsored children's science podcast, *Wow in the World* (Thomas and Raz, 2023).

**Figure 2.**
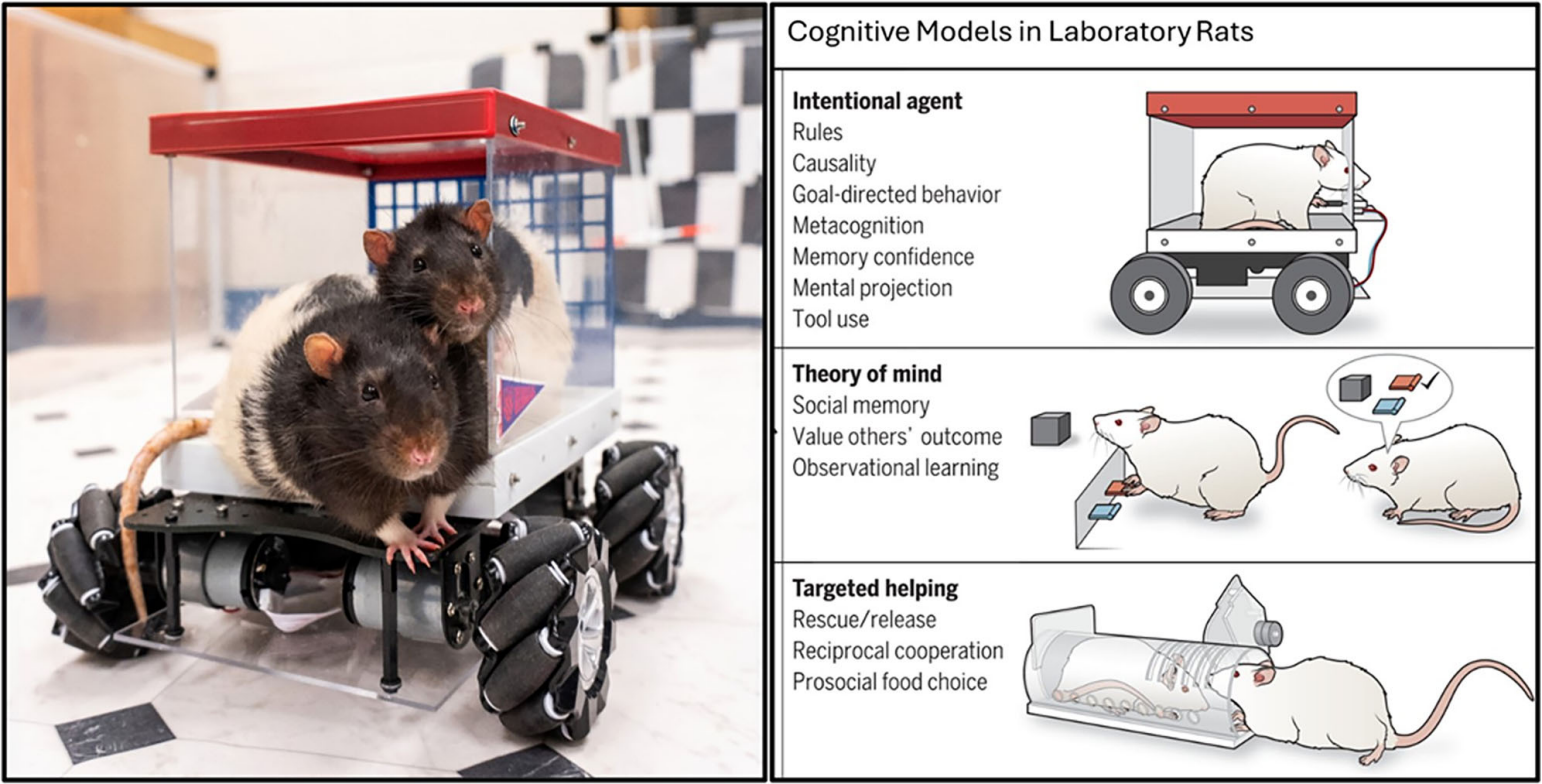
The driving rat project. Initially an exploratory endeavor, the rat-driving project yielded significant findings related to neuroplasticity, cognitive training, and enriched environments (left image). The project was later highlighted in *Science* as an example of using rodent models to investigate complex cognitive and emotional processes, including intentional agency [right image from Ben-Ami Bartal (2024). Reprinted with permission from AAAS].

Beyond media attention and science outreach, rodent-driving research has also influenced scientific discussions on animal cognition. Building on the concept of self-directed navigation in nonhuman animals, researchers have since trained goldfish to operate a small fish-operated vehicle (FOV), using strategic swimming patterns to navigate the fish car across terrestrial terrain toward a goal (Givon et al., 2022). This innovative study extends the idea that diverse species can adapt to novel movement-based tasks, further highlighting the flexibility of cognitive systems across taxa. Additionally, a recent *Science* review on neurobiological explorations of cognition and emotion in rodent models highlighted the driving rodent research as a model for investigating *Intentional Agency*—a cognitive category encompassing goal-directed behavior, mental projection, tool use, and metacognition (Ben-Ami Bartal, 2024; Fig. 2). This broad dissemination suggests that rodent-driving research has influenced both scientific and public views of rodent cognition.

Extending from the rodent driving research, our laboratory research took an unexpected turn when, during the quiet isolation of the pandemic, I began to notice a curious behavior in our driving-trained rats. As I entered the lab, I noticed that the rats would climb up and down the sides of their large enriched cages, extending their forepaws as if reaching toward me. Their behavior was reminiscent of my dog's excited behavior when asked if he wanted to take a walk. Whether the driving rats were excited by my presence or simply anticipating a Froot Loop reward, their enthusiasm was palpable and was consistent with what neuroscientists are currently characterizing as joy in nonhuman models (Nelson et al., 2023). This moment sparked a new question; specifically, could we systematically study the neurobiological underpinnings of positive anticipation?

Guided by the rats’ perceived enthusiasm—and emboldened by the curiosity-driven spirit of the driving rat project—we developed a new research protocol known as the Unpredictable Positive Event Responses (UPER) training program. This paradigm allows us to examine how extended anticipation of positive events influences emotional, cognitive, and behavioral outcomes. Our preliminary data suggest that UPER training fosters optimistic cognitive biases and enhances boldness in problem-solving tasks (Hartvigsen et al., 2024). As students and trainees in my lab embrace this new direction in research, they are discovering that a comprehensive understanding of anticipation for positive events, as well as other aspects of positive emotions, is fundamental to understanding the full array of neurobiological mechanisms and functions.

Reflecting on this journey, I see the driving rat project as a microcosm of scientific discovery—a playful question that evolved into a serious line of research, ultimately deepening our understanding of cognition, emotion, and learning in unexpected ways. Had I adhered to traditional constraints, this research might never have materialized. Echoing Panksepp's wisdom, the driving project serves as a reminder that curiosity is essential in pushing the boundaries of neuroscience.

## The Value of Messy Protocols and Evidence-Guided Exploration in Research Endeavors

In his 1950 article entitled *The Snark Was a Boojum*, neuroendocrinologist Frank Beach famously warned comparative psychologists and neuroscientists about the dangers of focusing too narrowly on laboratory-bred animals housed in pristine conditions. After analyzing studies published in the *Journal of Comparative Psychology*, Beach noted that, far from being comparative, most research focused almost exclusively on laboratory rats (Beach, 1950).

Seventy years later, we conducted an updated literature analysis, revealing that while rodents remain the dominant model, mice have now eclipsed rats in popularity—primarily due to their smaller size and the wealth of available genetic tools (Lambert et al., 2019). Whereas the predictability of laboratory environments and genetically similar animals offers experimental appeal, it may also represent a Faustian bargain for truth-seeking scientists. Beach further cautioned that embracing the inherent messiness of animal lives is crucial for understanding their true neural nature (Beach, 1950).

Recent research affirms Beach's concerns suggesting that using “squeaky clean” laboratory mice may undermine immune research. Pet-store mice, for example, with natural microbial exposures provide a more accurate model of the human immune system (Willyard, 2018). Similarly, sterile lab conditions and genetically cloned animals—often assumed to enhance biomedical research—may, in reality, compromise its translational power (Graham, 2021). Despite the practical advantages, rigidly controlled experimental conditions may strip research of the complexity required for true biological relevance.

**Figure 3.**
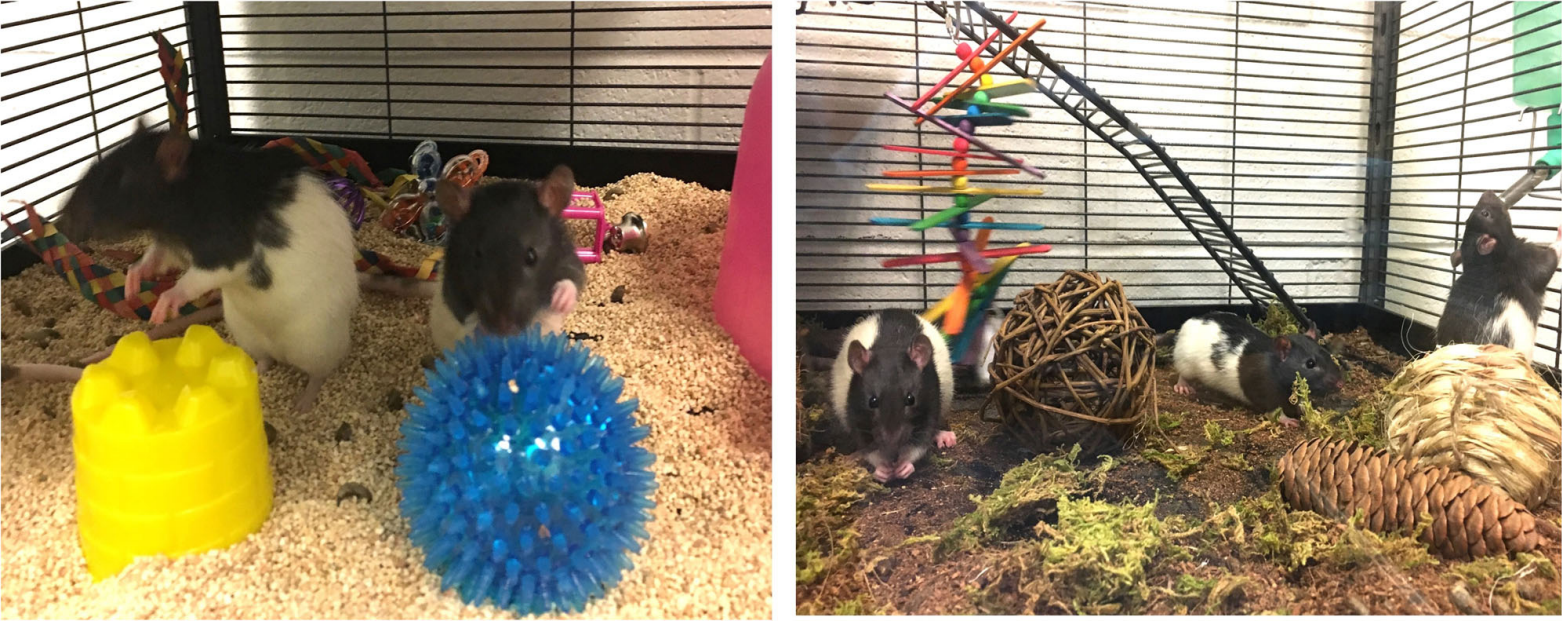
Artificial versus natural enriched environments. Rats exposed to standard enriched environments with manufactured components (left image) exhibit fewer markers of emotional resilience than rats raised in enriched environments with natural objects (right image).

Speaking of messy protocols, few experimental conditions in neuroscience have been as widely studied and debated as enriched environments. Our lab has taken this concept a step further, incorporating natural enriching elements—such as replacing conventional cage bedding with a sterile form of dirt and plastic balls with rocks (Lambert et al., 2015; Fig. 3). We observed markers of enhanced emotional resilience in animals housed in naturally enriched environments, as opposed to artificially enriched lab settings. Specifically, rats in naturalistic conditions exhibit higher DHEA-to-corticosterone fecal metabolite ratios, an endocrine marker associated with stress resilience in humans (Morgan et al., 2004), as well as adaptive coping mechanisms (Bardi et al., 2016).

A common criticism of enriched environment research is its lack of specificity—the difficulty of isolating the effects of individual variables within a dynamic setting. However, this perceived flaw may actually be a strength. The collective impact of multiple interacting variables may be far more influential than the deconstructed effects of individual stimuli. In this sense, enriched environment studies mirror the complexity and messiness of real-world neural adaptations.

Marian Diamond, one of the earliest pioneers in enriched environment research, faced significant resistance when she first presented the enriched environment findings. She and her colleagues reported that rats raised in complex environments exhibited thicker cortical areas—a claim that challenged the prevailing belief that postnatal brains were static and unchangeable. At a conference, an attendee dismissed Diamond's initial report of neuroplasticity, insisting that the adult brain could not change. Diamond's response? She simply stated that she had replicated the study and found consistent results, reinforcing the robustness of enriched environment effects (Diamond, 1988).

In a personal conversation, Marian Diamond encouraged my lab to explore the impact of naturally enriched habitats, a topic that she had investigated in a preliminary manner (Rosenzweig et al., 1972). Diamond's pioneering work with nontraditional enriched environments paved the way for a new understanding of experience-driven brain plasticity—a legacy that continues to shape research today. Thanks to the perseverance of enrichment researchers and other pioneers in this space, the impact of enriched environments is now widely recognized—from strengthening neural connections and cognitive flexibility to accelerating recovery from brain trauma (Baroncelli et al., 2010; Zentall, 2021; Han et al., 2022).

Enriched environment research may provide insights into the apparent disconnect between controlled laboratory research and real-world complexity, a disparity that may explain why a high percentage of pharmacological studies fail to translate to humans (Garner, 2014; Seyhan, 2019). Predictable laboratory conditions may contribute to this lack of translational efficacy. The wild rats we are currently investigating have very different life experiences than their laboratory counterparts. For example, wild rats have never lived in temperature- and light-controlled environments, nor have they ever existed outside the constant threat of predators and other life-threatening challenges. Thus, wild rat brains and bodies have adapted to a reality that no laboratory condition can fully replicate. If our goal is to improve neuroscience's translational success, one solution may lie in bringing laboratory rigor to the field—and real-world complexity into the lab (Smith, 2023). By carefully observing, recording, and interpreting neural responses in diverse, ecologically relevant settings, we can advance neuroscience in ways that are both methodologically rigorous and biologically meaningful.

Moving forward, we must embrace the inherent messiness of neuroscience while maintaining appropriate scientific rigor. As Frank Beach (1950) argued, we may need to sacrifice the *niceties* of tightly controlled laboratory settings in favor of conditions that better reflect real-world complexity. Evidence-guided exploration—rather than rigid, overly controlled methodologies—will yield a more accurate, nuanced, and ultimately translational understanding of the brain. Harkening back to Darwin's insights, the natural world, with all of its unpredictability and complexity, remains the most comprehensive and instructive laboratory available.

## Concluding with a Nod to “Once Upon a Time” Data

Embracing the full spectrum of scientific inquiry, including unconventional and exploratory research approaches, broadens our ability to develop meaningful interventions for psychiatric illnesses. With mental health disorders rising globally, particularly in the USA (Sterling and Platt, 2022), it is imperative to move beyond rigid experimental paradigms to uncover transformative insights into brain function and resilience. As described throughout the previous sections of this essay, history offers a roadmap for this approach. Darwin's journey was not confined to a sterile laboratory; his “living laboratory”—initially the Galápagos Islands and subsequently the surroundings of his Down House—provided the natural complexity necessary to reveal evolutionary truths. By embracing the dynamic, messy realities of nature, Darwin illuminated biological principles that had been hidden in the shadows for centuries.

Philosopher Nancy Cartwright underscores the importance of causal models that link theoretical frameworks to clinical outcomes, emphasizing the role of an “argument pyramid,” a structured approach that systematically integrates evidence from across the scientific literature (Cartwright and Hardie, 2012). These models highlight the critical point that exploratory research when guided by systematic observation and empirical validation has the power to produce transformative insights. It's important to recognize, however, that even carefully derived causal models and empirical findings are not static truths, as all findings are shaped by their specific context and time. This perspective aligns with the insights of Henry Donaldson, an early director of the Wistar Institute, the first major supplier of laboratory rats. Donaldson acknowledged that, even under tightly controlled conditions, rodent data were inherently “once upon a time data”; that is, rodent data were valid only within a specific context, time, and population (Logan, 1999). Thus, scientific progress depends on reasoned leaps of inference, as no finding exists in isolation from its context.

Considering the limitations of predictable, controlled experimental approaches, it's becoming increasingly clear that—like other high-stakes pursuits—playing it safe in neuroscience may carry the greatest risk. Accordingly, it's imperative to pass this perspective along to neuroscience students and trainees. As Darwin's *living laboratory* demonstrated, the most profound scientific breakthroughs do not emerge from certainty but from the courage to question, explore, and follow curiosity wherever it leads. Only by embracing this mindset can we illuminate the shadows of neuroscience and transform mental health outcomes for generations to come.

## Synthesis

Reviewing Editor: Christophe Bernard, INSERM &Institut de Neurosciences des Systèmes Decisions are customarily a result of the Reviewing Editor and the peer reviewers coming together and discussing their recommendations until a consensus is reached. When revisions are invited, a fact-based synthesis statement explaining their decision and outlining what is needed to prepare a revision will be listed below. The following reviewer(s) agreed to reveal their identity: NONE.

Thanks for this interesting piece

It resonates with my eNeuro editorial on "conditioning conformity". We, scientist, managed to impoverish our research remaining under the lamp post because that it is the only way to publish and get funded, since we collectively perpetuate this vicious circle (we review publications and grants).

## References

[B1] Bardi M, Kaufman C, Franssen C, Hyer MM, Rzucidlo A, Brown M, Tschirhart M, Lambert KG (2016) Paper or plastic? Exploring the effects of natural enrichment on behavioural and neuroendocrine responses in Long-Evans rats. J Neuroendocrinol 28. 10.1111/jne.12383 26970429

[B2] Barlow N (1958) The autobiography of Charles Darwin 1809-1882: with original omissions restored.

[B3] Baroncelli L, Braschi C, Spolidoro M, Begenisic T, Sale A, Maffei L (2010) Nurturing brain plasticity: impact of environmental enrichment. Cell Death Differ 17:1092–1103. 10.1038/cdd.2009.193 20019745

[B4] Beach FA (1950) The snark was a boojum. Am Psychol 5:115. 10.1037/h0056510

[B5] Ben-Ami Bartal I (2024) The complex affective and cognitive capacities of rats. Science 385:1298–1305. 10.1126/science.adq6217 39298607

[B6] Boulter M (2009) *Darwin’s garden: down house and the origin of species*. Berkeley CA: Counterpoint.

[B7] Buffenstein R, et al. (2022) The naked truth: a comprehensive clarification and classification of current “myths” in naked mole-rat biology. Biol Rev Camb Philos Soc 97:115–140. 10.1111/brv.12791 34476892 PMC9277573

[B8] Cait J, Cait A, Scott RW, Winder CB, Mason GJ (2022) Conventional laboratory housing increases morbidity and mortality in research rodents: results of a meta-analysis. BMC Biol 20:15. 10.1186/s12915-021-01184-0 35022024 PMC8756709

[B9] Carls-Diamante S (2022) Where is it like to be an octopus? Front Syst Neurosci 16:840022. 10.3389/fnsys.2022.840022 35401127 PMC8988249

[B10] Carter CS, DeVries AC, Getz LL (1995) Physiological substrates of mammalian monogamy: the prairie vole model. Neurosci Biobehav Rev 19:303–314. 10.1016/0149-7634(94)00070-H 7630584

[B11] Cartwright N, Hardie J (2012) *Evidence-based policy: a practical guide to doing it better*. New York: Oxford University Press.

[B12] Crawford LE, et al. (2020) Enriched environment exposure accelerates rodent driving skills. Behav Brain Res 378:112309. 10.1016/j.bbr.2019.112309 31629004

[B13] Darwin C (1872) *The expression of emotions in man and animals*. London: John Murray.

[B14] Darwin C (1964) On the origin of species by means of natural selection (Facsimile of the 1859 ed.). Boston: Harvard University Press. (Original work published 1859).

[B15] Diamond MC (1988) *Enriching heredity: the impact of the environment on the anatomy of the brain*. New York City: Free Press.

[B16] Dobzhansky T (1973) Nothing in biology makes sense except in the light of evolution. Am Biol Teach 35:125–129. 10.2307/4444260

[B17] Feynman RP (1955) The value of science. Eng Sci 19:13–15.

[B18] Feynman RP (1988) *What do you care what other people think? Further adventures of a curious character*. New York: W. W. Norton.

[B19] Garner JP (2014) The significance of meaning: why do over 90% of behavioral neuroscience results fail to translate to humans, and what can we do to fix it? ILAR J 55:438–456. 10.1093/ilar/ilu047 25541546 PMC4342719

[B20] Getz LL, Carter CS, Gavish L (1981) The mating system of the prairie vole, microtus ochrogaster: field and laboratory evidence for pair-bonding. Behav Ecol Sociobiol 8:189–194. 10.1007/BF00299829

[B21] Givon S, Samina M, Ben-Shahar O, Segev R (2022) From fish out of water to new insights on navigation mechanisms in animals. Behav Brain Res 419:113711. 10.1016/j.bbr.2021.113711 34896210

[B22] Graham AL (2021) Naturalizing mouse models for immunology. Nat Immunol 22:111–117. 10.1038/s41590-020-00857-2 33495644

[B23] Han Y, Yuan M, Guo Y-S, Shen X-Y, Gao Z-K, Bi X (2022) The role of enriched environment in neural development and repair. Front Cell Neurosci 16:890666. 10.3389/fncel.2022.890666 35936498 PMC9350910

[B24] Hartvigsen S, Shatalov B, Wixted J, Lambert K (2024) Altered cognitive, emotional and neuroplastic indices in a rat model of enhanced anticipation of appetitive events. Poster presented at the annual meeting of the Society for Neuroscience, Chicago IL.

[B25] Henrich J, Heine SJ, Norenzayan A (2010) The weirdest people in the world? Behav Brain Sci 33:61–83; discussion 83–135. 10.1017/S0140525X0999152X 20550733

[B26] Herculano-Houzel S (2009) The human brain in numbers: a linearly scaled-up primate brain. Front Hum Neurosci 3:31. 10.3389/neuro.09.031.2009 19915731 PMC2776484

[B27] Jacob J, et al. (2021) Cytoarchitectural characteristics associated with cognitive flexibility in raccoons. J Comp Neurol 529:3375–3388. 10.1002/cne.25197 34076254

[B28] Jacob J, et al. (2022) Divergent neural and endocrine responses in wild-caught and laboratory-bred rattus norvegicus. Behav Brain Res 432:113978. 10.1016/j.bbr.2022.113978 35753530

[B29] Jardim-Messeder D, Lambert K, Noctor S, Pestana FM, de Castro Leal ME, Bertelsen MF, Alagaili AN, Mohammad OB, Manger PR, Herculano-Houzel S (2017) Dogs have the most neurons, though not the largest brain: trade-off between body mass and number of neurons in the cerebral cortex of large carnivoran species. Front Neuroanat 11:118. 10.3389/fnana.2017.00118 29311850 PMC5733047

[B30] Lambert K (2018) *Well-grounded: the neurobiology of rational decisions*. New Haven, CT: Yale University Press.

[B31] Lambert K (2024) I taught rats to drive a car, and it may help us lead happier lives. The Conversation. Available at: https://theconversation.com/im-a-neuroscientist-who-taught-rats-to-drive-their-joy-suggests-how-anticipating-fun-can-enrich-human-life-239029

[B32] Lambert KG, Byrnes EM (2019) Challenges to the parental brain: neuroethological and translational considerations. Front Neuroendocrinol 53:100747. 10.1016/j.yfrne.2019.04.004 31004617

[B33] Lambert K, Kent M, Vavra D (2019) Avoiding Beach’s Boojum effect: enhancing bench to bedside translation with field to laboratory considerations in optimal animal models. Neurosci Biobehav Rev 104:191–196. 10.1016/j.neubiorev.2019.06.034 31278952

[B34] Lambert KG, Nelson RJ, Jovanovic T, Cerdá M (2015) *Brains in the city: neurobiological effects of urbanization*. Neurosci Biobehav Rev 58:107–122. 10.1016/j.neubiorev.2015.04.007 25936504 PMC4774049

[B35] Lázaro J, Hertel M, Sherwood CC, Muturi M, Dechmann DKN (2018) Profound seasonal changes in brain size and architecture in the common shrew. Brain Struct Funct 223:2823–2840. 10.1007/s00429-018-1666-5 29663134 PMC5995987

[B36] Logan CA (1999) The altered rationale for the choice of a standard animal in experimental psychology: Henry H. Donaldson, Adolf Meyer, and “the” albino rat. Hist Psychol 2:3–24. 10.1037/1093-4510.2.1.3 11623618

[B37] Merzenich MM, Van Vleet TM, Nahum M (2014) Brain plasticity-based therapeutics. Front Hum Neurosci 8:385. 10.3389/fnhum.2014.00385 25018719 PMC4072971

[B38] Morgan CA 3rd, Southwick S, Hazlett G, Rasmusson A, Hoyt G, Zimolo Z, Charney D (2004) Relationships among plasma dehydroepiandrosterone sulfate and cortisol levels, symptoms of dissociation, and objective performance in humans exposed to acute stress. Arch Gen Psychiatry 61:819–825. 10.1001/archpsyc.61.8.819 15289280

[B39] Nelson XJ, Taylor AH, Cartmill EA, Lyn H, Robinson LM, Janik V, Allen C (2023) Joyful by nature: approaches to investigate the evolution and function of joy in non-human animals. Biol Rev Camb Philos Soc 98:1548–1563. 10.1111/brv.12965 37127535

[B40] Panksepp J (2004) *Affective neuroscience: the foundations of human and animal emotions*. New York: Oxford University Press.

[B41] Panksepp J, Burgdorf J (2003) “Laughing” rats and the evolutionary antecedents of human joy? Physiol Behav 79:533–547. 10.1016/S0031-9384(03)00159-8 12954448

[B42] Raghanti MA, Spurlock LB, Treichler FR, Weigel SE, Stimmelmayr R, Butti C, Thewissen JG, Hof PR (2015) An analysis of von Economo neurons in the cerebral cortex of cetaceans, artiodactyls, and perissodactyls. Brain Struct Funct 220:2303–2314. 10.1007/s00429-014-0792-y 24852852

[B43] Roger M, Handasyde K (1999) *The koala*. Randwick, Sydney: UNSW Press.

[B44] Rosenzweig MR, Bennett EL, Diamond MC (1972) Brain changes in response to experience. Sci Am 226:22–29. 10.1038/scientificamerican0272-225062027

[B45] Seed A, Emery N, Clayton N (2009) Intelligence in corvids and apes: a case of convergent evolution? Ethology 115:401–420. 10.1111/j.1439-0310.2009.01644.x

[B46] Seyhan AA (2019) Lost in translation: the valley of death across preclinical and clinical divide – identification of problems and overcoming obstacles. Transl Med Commun 4:18. 10.1186/s41231-019-0050-7

[B47] Smith K (2023) Neuroscience goes wild. Nature 618:448–450. 10.1038/d41586-023-01926-w37433994

[B48] Sterling P, Platt ML (2022) Why deaths of despair are increasing in the US and not other industrial nations-insights from neuroscience and anthropology. JAMA Psychiatry 79:368–374. 10.1001/jamapsychiatry.2021.4209 35107578

[B49] Thomas G, Raz M (2023) Rat race [audio podcast episode]. Wow in the world. NPR. Available at: https://www.npr.org

[B50] Willyard C (2018) Squeaky clean mice could be ruining research. Nature 556:16–18. 10.1038/d41586-018-03916-9 29620765

[B51] Zentall TR (2021) Effect of environmental enrichment on the brain and on learning and cognition by animals. Animals 11:973. 10.3390/ani11040973 33807367 PMC8066627

